# The relationship between loneliness and social networking site addiction among Chinese college students: the roles of fear of missing out and the imaginary audience

**DOI:** 10.3389/fpsyg.2026.1792932

**Published:** 2026-04-14

**Authors:** Jiawei Guo, Yue Guo, Zhiqiang Jia, Meng Zhang

**Affiliations:** 1 School of Sociology, Beijing Normal University, Beijing, China; 2 School of Marxism Studies, Shanxi University, Taiyuan, China; 3 School of Digital Communication, Guangzhou Huashang College, Guangzhou, China; 4 School of Marxism, Central China Normal University, Wuhan, China

**Keywords:** Chinese college students, fear of missing out, imaginary audience, loneliness, longitudinal, social networking site addiction

## Abstract

**Objective:**

This study employed a three-wave longitudinal design to investigate whether fear of missing out (FoMO) mediates the relationship between loneliness and social networking site (SNS) addiction, and whether this indirect pathway is moderated by the imaginary audience (IA).

**Methods:**

A total of 1,337 Chinese college students (M_age = 19.86 years, SD = 1.79; 670 females) completed validated Chinese versions of measures assessing loneliness, FoMO, IA, and SNS addiction across three waves at six-month intervals. Regression-based mediation and moderation analyses were conducted using SPSS 26.0. Bootstrapping with 5,000 resamples was applied to test indirect effects, and simple slope analyses were performed for significant interactions.

**Results:**

T1 loneliness was positively associated with T3 SNS addiction (*β* = 0.19, *p* < 0.001). This association operated both directly (95% CI [0.14, 0.23]) and indirectly via T2 FoMO (95% CI [0.20, 0.28]), with the indirect effect accounting for 55.81% of the total effect (95% CI [0.39, 0.47]). T2 IA moderated the link between FoMO and SNS addiction (*β* = −0.07, *p* < 0.001). Contrary to expectations, the positive association was stronger among low-IA individuals.

**Conclusion:**

Interventions should target loneliness and FoMO while considering individual differences in IA.

## Introduction

The widespread use of social networking site (SNS) has provided unprecedented convenience for college students in learning, social interaction, and leisure activities. In China, mobile social applications such as WeChat, Weibo, QQ, and Xiaohongshu have become the dominant channels for communication. Almost all students report daily use, and a considerable proportion spend more than 4 h per day on these platforms (Cheng et al., 2022). Although such high levels of engagement are common and may in part reflect the functional role of SNS in maintaining social connections, they also raise concerns about students’ self-regulation difficulties when use becomes excessive or poorly controlled. Some individuals exhibit compulsive and maladaptive patterns of use that resemble SNS addiction (Chentsova et al., 2023; Han et al., 2025). SNS addiction is generally defined as compulsive online behavior characterized by an inability to effectively control the frequency and intensity of SNS use (Chen et al., 2018). In China, college students constitute the primary user group of smartphones and mobile social applications. Excessive use of these platforms may adversely affect their physical and mental health, including sleep disturbances, emotional problems, and impaired social functioning (Wang J. et al., 2025; Wang Y. et al., 2025). Consistent with recent perspectives on behavioral addictions, this pattern highlights the importance of a harm reduction approach, which emphasizes minimizing the negative consequences of excessive use while recognizing that SNS engagement itself is not inherently maladaptive (King et al., 2020). Therefore, identifying and understanding the underlying psychological mechanisms influencing SNS addiction among college students is importance for both prevention and intervention.

Among these mechanisms, loneliness has been consistently highlighted as a central factor. Loneliness, defined as a negative subjective emotional state arising from a discrepancy between desired and actual social relationships, has been widely conceptualized as a key psychosocial factor underlying SNS addiction. According to the cognitive–behavioral model of problematic internet use, maladaptive online behaviors often emerge from pre-existing psychosocial vulnerabilities (Davis, 2001). For college students experiencing loneliness—a subjective sense of social disconnection—difficulties in forming and maintaining satisfying offline relationships may generate negative beliefs about their social competence and unmet needs for belonging (Käll et al., 2020). These psychological deficits create a gap in social fulfillment, which students may attempt to address through SNS. The asynchronous, private, and interactive nature of these platforms provides opportunities for social connection and validation that are less accessible offline, encouraging compensatory use (Sharifpoor et al., 2017). A large-scale meta-analysis (82 studies, 90 independent samples, *N* = 48,383) demonstrated that SNS addiction and loneliness are weakly but significantly positively correlated (r = 0.052; Zhang et al., 2022). However, another meta-analysis of 32 studies with 26,166 participants revealed a stronger positive correlation between SNS addiction and loneliness (r = 0.21; Jing et al., 2025). The discrepancy in effect sizes suggests that the relationship may depend on factors such as the operationalization of addiction, cultural context, or patterns of social media engagement, highlighting the need for more nuanced investigation. To fill these gaps, this study adopted a three-wave longitudinal design to explore the relationship between loneliness and SNS addiction.

### Fear of missing out as a mediator

Building on the role of loneliness in SNS addiction, recent research has highlighted Fear of Missing Out (FoMO) as a complementary psychological mechanism. FoMO is defined as a pervasive anxiety experienced when individuals perceive that they are missing out on rewarding experiences or valuable information obtained by others in their absence (Przybylski et al., 2013). According to self-determination theory (SDT), the need for relatedness—the fundamental desire to feel connected to others and experience a sense of belonging—constitutes one of the three basic psychological needs essential for wellbeing (Ryan and Deci, 2017; Tandon et al., 2021). When this need is chronically frustrated, as in the case of intense loneliness, individuals experience a state of psychological deprivation that motivates them to seek compensatory avenues for social connection (Vansteenkiste and Ryan, 2013). College students are in a developmental period marked by heightened sensitivity to peer acceptance and social integration. For them, the frustration of relatedness needs may be particularly salient, amplifying their vigilance toward social cues and potential exclusion (Pi and Li, 2023). This vigilance manifests cognitively and affectively as FoMO—a persistent concern about being disconnected from the rewarding experiences of others (Bonfanti et al., 2023). Thus, loneliness, as an indicator of unmet relatedness needs, creates fertile ground for the emergence of FoMO.

A hallmark of FoMO is the persistent concern with staying informed about others’ activities. Features of SNS, such as real-time updates, status sharing, and interactive content, provide a readily accessible environment to satisfy this desire for social information (Wang J. et al., 2025; Wang Y. et al., 2025). Consequently, individuals with higher levels of FoMO are more likely to engage extensively with social media, increasing the risk of compulsive or maladaptive patterns of use. Supporting this perspective, empirical studies have consistently identified FoMO as a significant predictor of SNS addiction, with higher FoMO levels associated with more frequent and problematic social media engagement (Cui et al., 2024; Tseng and Huang, 2025).

### Imaginary audience as a moderator

More recently, researchers have begun to explore boundary conditions that may shape the strength of the FoMO–addiction relationship. One such factor is the concept of the imaginary audience (IA), which originates from Elkind (1967) theory of adolescent egocentrism. IA refers to the tendency to believe that one’s behaviors and appearance are the focus of others’ attention and are constantly being evaluated. It reflects a difficulty in differentiating between one’s own preoccupations and those of others (Cingel and Krcmar, 2014). Although initially conceptualized as a developmental phenomenon of adolescence, research suggests that IA may persist into emerging adulthood, particularly in contexts that amplify self-consciousness and social visibility (Kreyszig, 2006).

In the context of social media, the architectural features of platforms—such as visible audiences, quantifiable feedback (e.g., likes and views), and permanent records of one’s activities—may uniquely amplify IA tendencies (Ranzini and Hoek, 2017). For individuals high in IA, the online environment transforms abstract beliefs about being watched into concrete, perceived evidence of social scrutiny. When such individuals experience FoMO, this anxiety may be intensified by concerns about how their absence from social activities might be perceived by others (Takishima-Lacasa et al., 2014). In other words, high-IA individuals are not only afraid of missing out; they are also afraid of being seen as missing out, or of being judged negatively for their social absence. This dual concern may more strongly compel them to engage compulsively with SNS to maintain social visibility and manage others’ impressions.

In contrast, individuals with low IA are generally less preoccupied with others’ perceptions and social evaluation (Ranzini and Hoek, 2017). For them, FoMO may remain a relatively contained anxiety about missing experiences, without the added pressure of imagined social judgment, and thus may be less likely to translate into compulsive, addictive patterns of use. From this perspective, IA functions as a cognitive amplifier—a boundary condition that shapes the strength of the translation from FoMO to addictive behavior.

Furthermore, considering the cultural context of the present study, IA may hold particular relevance among Chinese college students. Chinese culture, rooted in collectivist values, places strong emphasis on social harmony, face concern (mianzi), and sensitivity to others’ evaluations (Oyserman et al., 2002). These cultural orientations may heighten individuals’ attunement to social visibility and external judgment, potentially making IA a more salient cognitive pattern in this population. Thus, examining IA as a moderator within a Chinese sample provides a culturally grounded extension of the theoretical framework.

### The current study

Although previous studies have linked loneliness to SNS addiction, the longitudinal associations between these variables and their underlying mechanisms, remain insufficiently understood. In particular, little research has examined how loneliness predicts changes in SNS addiction over time or has integrated FoMO and IA within a unified longitudinal framework. Moreover, most existing evidence is based on Western samples, with limited attention to Chinese college students, who are highly embedded in mobile social networking contexts.

Drawing on self-determination theory and the cognitive–behavioral perspective, the present study employed a three-wave longitudinal design to examine a moderated mediation model. Specifically, the current study tested whether loneliness prospectively predicts SNS addiction, whether FoMO mediates this longitudinal association, and whether IA moderates the link between FoMO and SNS addiction. Individuals experiencing unmet relatedness needs may develop heightened FoMO, which increases compulsive engagement with SNS. This effect may be stronger among individuals with higher levels of IA due to their heightened self-consciousness and perceived social visibility.

Accordingly, we hypothesized that:

*H1*: Loneliness positively predicts SNS addiction.

*H2*: FoMO mediates the relationship between loneliness and SNS addiction.

*H3*: IA moderates the association between FoMO and SNS addiction, such that the association is stronger at higher levels of IA.

## Methods

### Participants

This study employed a convenience sampling strategy to recruit undergraduate students from a university in Shanxi Province, China. The first wave of data collection (T1) was conducted in September 2024, yielding 1,400 valid questionnaires. The second wave (T2) took place in December 2024, during which 1,361 participants from the T1 sample were successfully followed up and completed the survey. The third wave (T3) was completed in March 2025, with 1,337 participants completing all three waves of data collection, constituting the final longitudinal sample. Among these participants, 670 were female, and the mean age was 19.86 years (SD = 1.79). Of the participants, 63.4% were only children, and 67.5% reported a rural background. To examine whether sample attrition introduced systematic bias, independent-samples t-tests were conducted to compare participants who dropped out with those who remained in the study on the core variables including loneliness, FoMO, IA, and SNS addiction (all *ps*>0.05). The results indicated no statistically significant differences between the two groups on any of these variables, suggesting that sample attrition did not exert a systematic influence on the study findings. All participants were informed of the voluntary and anonymous nature of the study, and written informed consent was obtained prior to participation. The study was conducted in accordance with the Declaration of Helsinki, and ethical approval was obtained from the university’s Institutional Review Board (Approval No. 20240513).

## Measures

### Loneliness

Loneliness was assessed using the UCLA Loneliness scale developed by Hays and DiMatteo (1987). The scale consists of 8 items rated on a 7-point Likert scale (1 = not at all characteristic, 7 = extremely characteristic). A sample item is, “Lacking the company of others.” Higher scores indicate greater levels of loneliness. In the present study, the scale demonstrated excellent internal consistency (Cronbach’s *α* = 0.94).

### Fear of missing out

FoMO was measured using the FoMO scale developed by Przybylski et al. (2013). The scale contains 10 items rated on a 5-point Likert scale (1 = not at all true of me, 5 = extremely true of me). A sample item is, “I fear my friends have more rewarding experiences than me.” Higher mean scores reflect greater FoMO. The internal consistency in this study was good (Cronbach’s *α* = 0.92).

### Imaginary audience

IA was assessed with the Imaginary Audience subscale of the Egocentrism Scale developed by Liu and Wu (2010). The subscale consists of 6 items rated on a 5-point Likert scale (1 = strongly disagree, 5 = strongly agree). A sample item is, “It would be very embarrassing to trip and fall in a public place.” Higher scores indicate stronger imaginary audience beliefs. The internal consistency coefficient in this study was acceptable (Cronbach’s *α* = 0.90).

### Social networking site addiction

SNS addiction was measured using the Weixin Intrusion scale developed by Hou (2017). The measure consists of 10 items rated on a 5-point Likert scale (1 = never, 5 = always). A sample item is, “I often think about WeChat when I am not using it.” Higher scores indicating greater SNS addiction. In the current study, the scale showed good reliability (Cronbach’s α = 0.91).

### Data analysis

All statistical analyses were conducted using SPSS 26.0. Descriptive statistics and Pearson correlations were first computed to examine associations among loneliness, FoMO, IA, and SNS addiction. Mediation was tested using PROCESS macro (Model 4) with 5,000 bootstrap resamples, and moderation was tested using PROCESS (Model 14). All variables were mean-centered prior to the creation of interaction terms. Simple slope analyses were performed to further probe significant interaction effects. Statistical significance was set at *p* < 0.05 for all analyses.

## Results

### Common method Bias test

To examine the potential common method bias, Harman’s single-factor test was conducted. The results indicated that 34 factors had eigenvalues greater than 1, with the first factor accounting for 29.21% of the variance, which is below the critical threshold of 40% (Podsakoff et al., 2003). These results suggest that common method bias was not a concern in the present study.

### Descriptive statistics and correlations

Descriptive statistics and correlation analyses showed that all study variables were positively correlated in the expected directions (see Table 1). Specifically, T1 loneliness was positively correlated with T2 FoMO, T2 IA, and T3 SNS addiction. Similarly, T2 FoMO was positively correlated with both T2 IA and T3 SNS addiction, while T2 IA was also positively associated with T3 SNS addiction. These results provided preliminary support for the hypothesized relationships.

**Table 1:** Variable descriptions and correlations.

Variables	*M*(*SD*)	1	2	3	4
1. T1 Loneliness	18.38 (10.70)	1			
2. T2 Fear of missing out	20.67 (9.06)	0.58^**^	1		
3. T2 Imaginary Audience	14.08 (5.75)	0.54^**^	0.64^**^	1	
4. T3 Social Network Sites Addiction	19.60 (8.60)	0.43^**^	0.53^**^	0.51^**^	1

^**^p < 0.01.

### Mediating role of FoMO in the link between loneliness and SNS addiction

A mediation analysis was conducted to examine whether T2 FoMO mediated the association between T1 loneliness and T3 SNS addiction. As shown in Table 2, T1 loneliness significantly influenced T2 FoMO (*β* = 0.56, *p* < 0.001), and T2 FoMO significantly influenced T3 SNS addiction (*β* = 0.42, *p* < 0.001). The direct effect of T1 loneliness on T3 SNS addiction remained significant (*β* = 0.19, *p* < 0.001). The indirect effect via T2 FoMO was 0.24, accounting for 55.81% of the total effect (0.43) see Table 3. Bootstrap analyses with 5,000 resamples confirmed that the 95% confidence interval (CI) of the indirect effect did not include zero, indicating that the longitudinal mediation effect was significant.

**Table 2:** Mediation model testing and coefficient summary.

Variables	T2 fear of missing out		T3 social network sites addiction	
	*β*	*t*	*β*	*t*
T1 Loneliness	0.56	29.75^***^	0.19	8.35^***^
T2 Fear of missing out			0.42	18.74^***^
*R^2^*	0.31		0.31	
*F*	884.81^***^		431.67^***^	

^***^p < 0.001.

**Table 3:** Regression coefficients for mediated relationships.

Model pathways	Estimate	*SE*	Bootstrapping 95% CI	
			Lower	Higher
Total effect	0.43	0.02	0.39	0.47
Direct effect	0.19	0.02	0.14	0.23
Indirect effect	0.24	0.02	0.20	0.28

### Moderating role of IA in the link between FoMO and SNS addiction

We further examined whether T2 IA moderated the association between T2 FoMO and T3 SNS addiction. As shown in Table 4, after controlling for T1 loneliness, the interaction term FoMO × IA was significant (*β* = −0.07, *p* < 0.001), suggesting the moderating effect of IA.

**Table 4:** Moderated mediation analysis summary.

Predictor variables	Outcome variables			
	T2 fear of missing out		T3 social network sites addiction	
	*β*	*t*	*β*	*t*
T1 Loneliness	0.56	29.69^***^	0.12	5.34^***^
T2 Fear of missing out			0.33	12.70^***^
T2 Fear of missing out × T2 Imaginary Audience			−0.07	−4.01^***^
*R^2^*	0.31		0.34	
*F*	881.23^***^		254.32^***^	

^***^p < 0.001.

**Figure 1:**
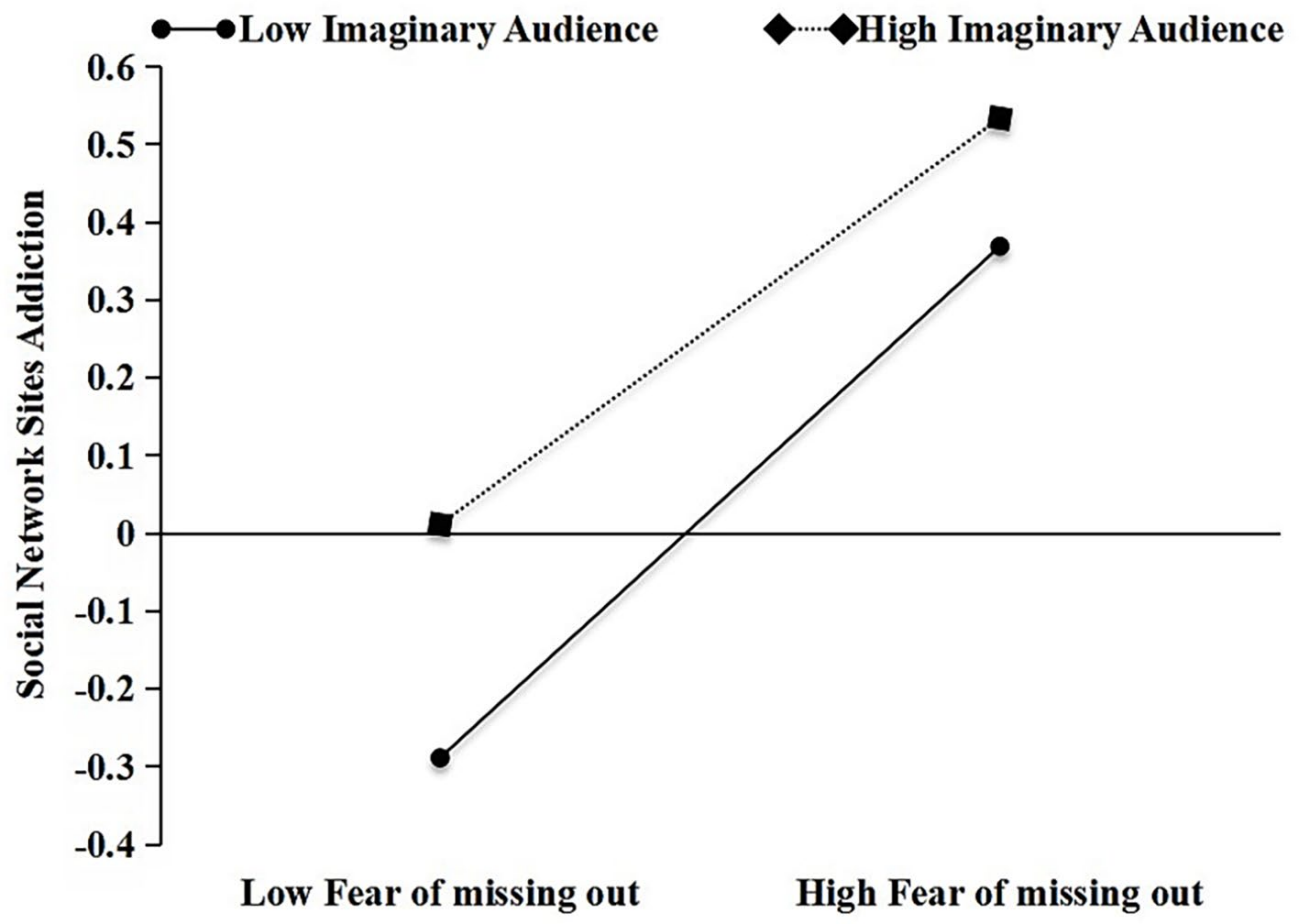
Simple slope analysis.

Simple slope analysis (see Figure 1) revealed that FoMO was positively associated with SNS addiction at both high and low levels of IA. However, the effect was stronger for individuals with low IA (simple slope = 0.40, *p* < 0.001) than for those with high IA (simple slope = 0.26, *p* < 0.001), suggesting that higher IA attenuates the impact of FoMO on SNS addiction. These findings partially support Hypothesis 3; however, the direction of the moderation was opposite to the initial prediction.

## Discussion

The present study adopted a one-year, three-wave longitudinal design to examine the pathways linking loneliness to SNS addiction among Chinese college students. We specifically focused on the mediating role of FoMO and the moderating role of IA. Overall, the findings extend prior research by demonstrating (a) a robust positive longitudinal association between loneliness and SNS addiction, (b) an indirect pathway through FoMO, and (c) an unexpected moderating effect of IA that challenges conventional assumptions.

Consistent with H1 and prior meta-analyses and longitudinal investigations (Wang J. et al., 2025; Wang Y. et al., 2025), loneliness emerged as a positive predictor of SNS addiction. Individuals experiencing higher levels of loneliness were more likely to engage in maladaptive patterns of SNS use. These results support the cognitive–behavioral model of problematic internet use, which posits that deficits in offline social relationships foster maladaptive cognitions and unmet belonging needs. These deficits, in turn, drive compensatory engagement in online environments (Bai et al., 2025; Xu and He, 2025).

From a theoretical perspective, these findings underscore the enduring relevance of self-determination theory. When the fundamental need for relatedness is unmet, individuals may turn to digital platforms in search of social connection and emotional validation (Liang et al., 2026). However, reliance on SNS often fails to satisfy deep social needs, potentially exacerbating compulsive usage in a self-perpetuating cycle (Fam and Männikkö, 2025). Our study situates this relationship within the Chinese college student population, which is characterized by high social media engagement and developmental vulnerability in social identity formation. This contextualization highlights the cultural and developmental specificity of SNS addiction. Online engagement may serve as a readily available but ultimately insufficient substitute for meaningful offline interactions.

Supporting Hypothesis 2, the present study demonstrates that FoMO mediates the relationship between loneliness and SNS addiction. Specifically, loneliness was associated with higher levels of FoMO, which in turn predicted greater SNS addiction (Gao et al., 2024; Li et al., 2023). This suggests that FoMO operates as a proximal cognitive–affective response to perceived social disconnection, translating distal feelings of loneliness into immediate motivations for online engagement (Elhai et al., 2025).

Integrating this perspective with the self-determination theory, the results suggest that unmet needs for relatedness manifest as heightened vigilance toward social information, which may lead to maladaptive online behaviors (Zhang, 2025). By demonstrating this indirect pathway, the study positions FoMO as a central psychological mechanism in problematic digital use (Rozgonjuk et al., 2020; Şahin et al., 2025). When the need for relatedness is thwarted, individuals may become preoccupied with others’ social activities and opportunities for social inclusion. This heightened vigilance is captured by FoMO, which reflects concerns about being excluded from rewarding social experiences. Thus, FoMO translates loneliness into an immediate motivational state with a perceived urgency to remain socially connected. SNS continuously provide cues about others’ activities, making them particularly compelling under such conditions.

The mediating role of FoMO clarifies how loneliness contributes to SNS addiction. This finding refines self-determination–based accounts of problematic social media use by specifying FoMO as the cognitive–motivational mechanism linking relatedness need frustration to maladaptive online behavior. These results highlight the importance of addressing FoMO-related cognitions in interventions targeting SNS addiction, particularly for individuals experiencing social isolation. However, it is worth noting that unmeasured confounders such as depression or anxiety may partially inflate this mediated pathway. Individuals experiencing depressive symptoms often exhibit both heightened loneliness and increased sensitivity to social exclusion, which could amplify FoMO and subsequent SNS use. Future research should statistically control for these variables to isolate the unique effect of loneliness on SNS addiction via FoMO.

Partially supporting H3, IA attenuated rather than amplified the association between FoMO and SNS addiction. The moderating coefficient of IA was *β* = −0.07 (*p* < 0.001), indicating a weak but significant negative moderating effect on the relationship between FoMO and SNS addiction. Specifically, for each one-unit increase in IA level, the positive predictive effect of FoMO on SNS addiction decreased by 0.07 units.

Although individuals high in IA exhibited higher baseline levels of SNS addiction, the slope of the FoMO–addiction relationship was steeper among those low in IA. Simple slope analysis revealed that for individuals with low IA, the effect of FoMO on SNS addiction was significant (*β* = 0.40, *p* < 0.001), whereas for individuals with high IA, this effect was substantially weaker (*β* = 0.26, *p* < 0.001). This counterintuitive pattern may reflect several mechanisms.

First, a ceiling effect may be present: high-IA individuals already demonstrate elevated SNS engagement, leaving limited scope for FoMO to further influence addictive behaviors. This interpretation is consistent with the correlation results in Table 1, which show a significant positive correlation between IA and SNS addiction (r = 0.51, *p* < 0.01), suggesting that high-IA individuals have already reached a relatively high baseline level of SNS use.

Second, heightened IA in the Chinese cultural context may reflect elevated self-consciousness and social monitoring, which paradoxically constrains compulsive SNS use. Within the collectivistic cultural framework, individuals with high IA are particularly attuned to social evaluation and face concerns (mianzi). While IA shares conceptual ground with social anxiety and fear of negative evaluation, the positive correlation between IA and SNS addiction (r = 0.51) supports a strategic regulation interpretation rather than social avoidance—high-IA individuals remain highly engaged online but regulate their use to manage impressions. This heightened sensitivity may lead them to strategically regulate their online behaviors to avoid negative social judgment, thereby weakening the driving effect of FoMO on compulsive SNS use. In contrast, individuals with low IA are less preoccupied with others’ perceptions and social visibility. Lacking this self-regulatory mechanism, they may be more likely to engage in excessive SNS use as a direct response to FoMO-related anxiety, resulting in a stronger predictive effect of FoMO on their SNS addiction.

These findings align with recent theoretical work highlighting IA’s dual role. It can function as both a risk factor and a protective factor depending on contextual and individual differences (Shi et al., 2024; Sun et al., 2024). In collectivistic, socially evaluative contexts, IA may promote strategic online engagement that mitigates FoMO-driven compulsivity. However, it is worth noting that personality traits such as neuroticism or public self-consciousness may confound the observed moderating effect of IA. Individuals high in neuroticism tend to be more vigilant toward social threats and may therefore exhibit both heightened IA and stronger FoMO–addiction associations. The inclusion of such traits in future models would help clarify whether IA exerts a unique moderating effect beyond broader dispositional tendencies. Additionally, while the present study modeled the moderating effect of IA as linear, it is possible that nonlinear or threshold patterns exist—for instance, the effect of FoMO on SNS addiction may plateau beyond a certain level of IA. Future research with larger samples could explore such nonlinear dynamics to further refine understanding of IA’s boundary conditions. By demonstrating that IA may elevate baseline addiction risk while dampening the effect of FoMO, the study provides a more nuanced understanding of IA’s role in digital behavior.

### Alternative pathways and directionality

The present study tested a unidirectional model in which loneliness predicts FoMO, which in turn predicts SNS addiction, consistent with self-determination theory’s emphasis on unmet relatedness needs. However, alternative directional pathways are theoretically plausible. For example, higher levels of SNS addiction may increase FoMO through exposure to social comparisons and curated peer content, which could subsequently exacerbate loneliness (Koçak et al., 2025). Reciprocal dynamics may also operate, wherein loneliness and SNS addiction mutually reinforce each other over time through a feedback loop (Hu and Xiang, 2024). Although our three-wave longitudinal design establishes temporal precedence (loneliness at T1, FoMO at T2, SNS addiction at T3), it cannot rule out reverse or bidirectional effects. Future research using cross-lagged panel models or random-intercept cross-lagged panel models is needed to systematically examine these competing pathways and provide a more comprehensive understanding of the dynamic interplay among loneliness, FoMO, and SNS addiction.

### Limitations

Several limitations warrant consideration. First, although the present study employed a three-wave longitudinal design, causal inferences remain tentative. The time intervals between waves may not fully capture the dynamic processes among the variables. Moreover, while IA was measured concurrently with FoMO at T2, prior to the T3 assessment of SNS addiction, this design preserves temporal precedence of the predictor relative to the outcome. IA is conceptualized as a relatively stable trait-like characteristic, which minimizes potential state-induced confounding. Future studies using fully lagged designs with IA measured at earlier waves could provide stronger evidence for its moderating effect and clarify causal inference. Second, reliance on self-report measures introduces potential biases, such as social desirability and shared method variance. Incorporating behavioral data or multi-informant assessments could strengthen future research. Third, convenience sampling from a single university limits generalizability. Replication across diverse institutions and cultural contexts is needed. Fourth, potential unmeasured confounders, such as depression, anxiety, or personality traits, may partially account for the observed associations. Future research should adopt a more comprehensive model controlling for these variables.

### Implications

Theoretically, this study advances current models of SNS addiction by integrating loneliness, FoMO, and IA into a comprehensive framework. Loneliness functions as a distal risk factor, FoMO as a proximal psychological mechanism, and IA as a boundary condition shaping the strength of these associations. This multi-level perspective contributes to a more sophisticated understanding of the psychosocial factors underlying SNS addiction.

Practically, the findings underscore the importance of interventions targeting loneliness and unmet relatedness needs among college students. Cognitive–behavioral approaches promoting offline social engagement and reducing FoMO-related anxiety may be particularly effective. Psychoeducational programs addressing IA tendencies could support students in managing self-consciousness and perceived social scrutiny, fostering healthier online behaviors. Moreover, interventions can also focus on improving the quality of SNS use, rather than solely reducing usage. For example, fostering more intentional and self-regulated engagement patterns, alongside efforts to reduce maladaptive social comparison while maintaining meaningful social connection, may support more balanced and adaptive SNS use.

## Conclusion

The present study examined the link between loneliness and SNS addiction. Results indicated that loneliness was positively associated with SNS addiction. Moreover, fear of missing out mediated this association, and the effect was moderated by imaginary audience. Furthermore, the effect of fear of missing out on SNS addiction was stronger among individuals with lower levels of imaginary audience.

## Data Availability

The raw data supporting the conclusions of this article will be made available by the authors, without undue reservation.
