# Correction: The relationship between loneliness and social networking site addiction among Chinese college students: the roles of fear of missing out and the imaginary audience

**DOI:** 10.3389/fpsyg.2026.1986511

**Published:** 2026-10-06

**Authors:** Jiawei Guo, Yue Guo, Zhiqiang Jia, Meng Zhang

**Affiliations:** 1 School of Sociology, Beijing Normal University, Beijing, China; 2 School of Marxism Studies, Shanxi University, Taiyuan, China; 3 School of Digital Communication, Guangzhou Huashang College, Guangzhou, China; 4 School of Marxism, Central China Normal University, Wuhan, China

**Keywords:** Chinese college students, fear of missing out, imaginary audience, loneliness, longitudinal, social networking site addiction

There was a mistake in Table 1 as published. The means and standard deviations reported for T1 Loneliness, T2 Fear of Missing Out, T2 Imaginary Audience, and T3 Social Networking Sites Addiction were incorrect. The corrected Table 1 appears below.

**Table 1:** Variable descriptions and correlations.

Variables	*M* (SD)	1	2	3	4
1.T1 loneliness	18.38 (10.70)	1			
2.T2 fear of missing out	20.67 (9.06)	0.58^**^	1		
3.T2 imaginary audience	14.08 (5.75)	0.54^**^	0.64^**^	1	
4.T3 social network sites addiction	19.60 (8.60)	0.43^**^	0.53^**^	0.51^**^	1

^**^p < 0.01.

The **Ethics statement** was erroneously given as

The studies involving humans were approved by the Shanxi University's Institutional Review Board. The studies were conducted in accordance with the local legislation and institutional requirements. The participants provided their written informed consent to participate in this study.

A correction has been made to the approving institution and study procedures. The corrected **Ethics statement** appears below:

The studies involving humans were approved by the Ethical Review Committee of the School of Physical Education and Sport, Beijing Normal University (Approval No. BNU-TY-20240721-1). The studies were conducted in accordance with the Declaration of Helsinki. The participants provided their written informed consent to participate in this study.

In the **Abstract**, the time point of the Imaginary Audience variable was incorrectly reported as T3. This has been corrected to read:

T2 IA moderated the link between FoMO and SNS addiction (β = −0.07, *p* < 0.001).

Two section headings in the **Results** section were incorrectly reported in the published version.

The original heading was “Mediating role of rumination in the link between cybervictimization and sleep quality”.

The corrected heading is “Mediating role of FoMO in the link between loneliness and SNS addiction”.

The original heading was “Moderating role of self-compassion in the cybervictimization–rumination link”.

The corrected heading is “Moderating role of IA in the link between FoMO and SNS addiction”

The description and citation of the loneliness measure were incorrectly reported in the published article. The measure was mistakenly described as the 20-item UCLA Loneliness Scale developed by Russell et al. (1980), whereas the study used the 8-item version developed by Hays and DiMatteo (1987).

A correction has been made to the **Measures** section

“Loneliness was assessed using the UCLA Loneliness scale developed by Hays and DiMatteo (1987). The scale consists of 8 items rated on a 7-point Likert scale (1 = not at all characteristic, 7 = extremely characteristic). A sample item is, “Lacking the company of others.” Higher scores indicate greater levels of loneliness. In the present study, the scale demonstrated excellent internal consistency (Cronbach's α = 0.94).”

The reference for Russell, D., Peplau, L. A., & Cutrona, C. E. (1980). The revised UCLA Loneliness Scale: Concurrent and discriminant validity evidence. Journal of Personality and Social Psychology, 39(3), 472–480. doi: 10.1037/0022-3514.39.3.472, which was cited in connection with the loneliness measure in the published article, was incorrect.

It should be Hays, R. D., and DiMatteo, M. R. (1987). A short-form measure of loneliness. *J. Pers. Assess*. 51, 69–81. doi: 10.1207/s15327752jpa5101_6

The original version of this article has been updated.

